# Iron Overload, Clonal Hematopoiesis, and Cancer Risk in Aging and Transfusion-Dependent Populations: A Literature Review

**DOI:** 10.7759/cureus.85936

**Published:** 2025-06-13

**Authors:** Quang D La, Marc Faltas, Armin Zavareh, Zarnum Gul, Uzair Uzzi, Jasneel S Kahlam, Aiman Baloch, Nehal Revuri, Shanmukh Bachhu, Francis Pryor, Sobia Ahmed, Muhammad Ayub

**Affiliations:** 1 Medicine, The Innovative STEMagazine, College Station, USA; 2 Medicine, Rowan-Virtua School of Osteopathic Medicine, Stratford, USA; 3 Medicine, Mekran Medical College, Turbat, PAK; 4 Internal Medicine, Stony Brook Southampton, Hackettstown, USA; 5 Neurological Surgery, The Innovative STEMagazine, College Station, USA; 6 Civil Engineering, University of California, Berkeley, Berkeley, USA; 7 Medicine, Lake Erie College of Osteopathic Medicine, Erie, USA; 8 Radiology, Bolan Medical Complex Hospital, Quetta, PAK

**Keywords:** cancer, chip, clonal hematopoiesis, genomic instability, iron homeostasis, iron metabolism, iron overload, myelodysplastic syndromes, risk of cancer, transfusion-dependent groups

## Abstract

Aging also contributes to cancer risk factor potentiation by disturbed iron metabolism and genomic instability, both of which contribute to enhanced risk of cancer, particularly in transfusion-dependent groups such as patients with β-thalassemia or myelodysplastic syndromes. Systemic iron overload results from chronic transfusions and progressively disturbed iron homeostasis and clonal hematopoiesis of indeterminate potential (CHIP) that contribute to oncogenic burden. All these create a permissive profile in which carcinogenesis is favored by oxidative stress, mitochondrial dysfunctions, immune suppression, and disrupted DNA repair.

This review synthesizes current literature regarding iron overload, clonal hematopoiesis, and aging to examine the combined impact on initiation of cancer (Appendices). It evaluates processes, such as Fenton chemistry, reactive oxygen species (ROS)-mediated DNA damage, pro-inflammatory signals, and hematopoietic clonal expansion, and therapeutic options, such as iron chelation, risk monitoring, and age-targeted therapies in risk-carrying elderly groups.

Iron overload in aging and transfusional individuals is characterized by high ferritin, augmented non-transferrin-bound iron, and oxidative DNA damage, which all raise the risk of cancer, especially hepatocellular carcinoma. Concurrently, clonal hematopoiesis of indeterminate potential (CHIP) increases with age and predisposes individuals to hematologic malignancies and cardiovascular disease. The interaction of these factors increases mutagenesis and inflammation. Iron chelation therapy (ICT) has been found to be effective in the reduction of iron burden and prevention of complications, but side effects and compliance are problematic. Some new evidence suggests that individualized ICT, combined with CHIP screening and non-invasive imaging (e.g., T2* MRI), can prevent malignancy in high-risk patients.

Iron overload in aging and transfusion-dependent populations is a critical, modifiable risk factor for cancer. The accumulation of effects of clonal hematopoiesis underscores the need to incorporate monitoring and intervention strategies. Future research has to define molecular targets in iron and hematopoietic networks to employ individualized therapies that reduce the emergence of cancer and increase health span in aging, vulnerable populations.

## Introduction and background

**Figure 1:**
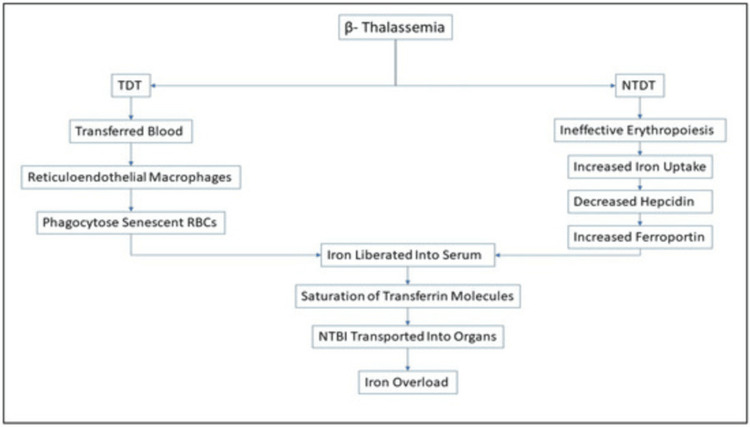
Schematic representation of the iron accumulation mechanisms in transfusion-dependent (TDT) and non-transfusion-dependent (NTDT) β-thalassemia by Basu et al., 2023. Ineffective erythropoiesis leads to iron dysregulation in both patient groups, but in TDT, the necessity for repeated transfusions leads to more rapid and severe iron overload than in NTDT. Image by Basu et al., 2023 [4]. Reproduced with open access permissions under the terms and conditions of the Creative Commons Attribution (CC BY) license (https://creativecommons.org/licenses/by/4.0/).

Iron overload is a serious complication of transfusion-dependent anemias, such as β-thalassemia major, where each unit of blood transfused loads the body with 200-250 mg of iron, for which there is no physiological mechanism in the human body to excrete this excess iron, and it is stored in critical organs [1]. This storage causes oxidative stress and DNA damage, which, in turn, confers an increased risk of malignancy, namely hepatocellular carcinoma [2]. Borgna-Pignatti et al. reported that iron overload-induced liver damage is one of the leading causes of death in thalassemia major patients, accentuating the carcinogenic potential of iron overload [3]. The difference between the rate and extent of iron accumulation in transfusion-dependent and non-transfusion-dependent individuals with β-thalassemia has been well illustrated under different models (Figure 1) [4].

**Figure 2:**
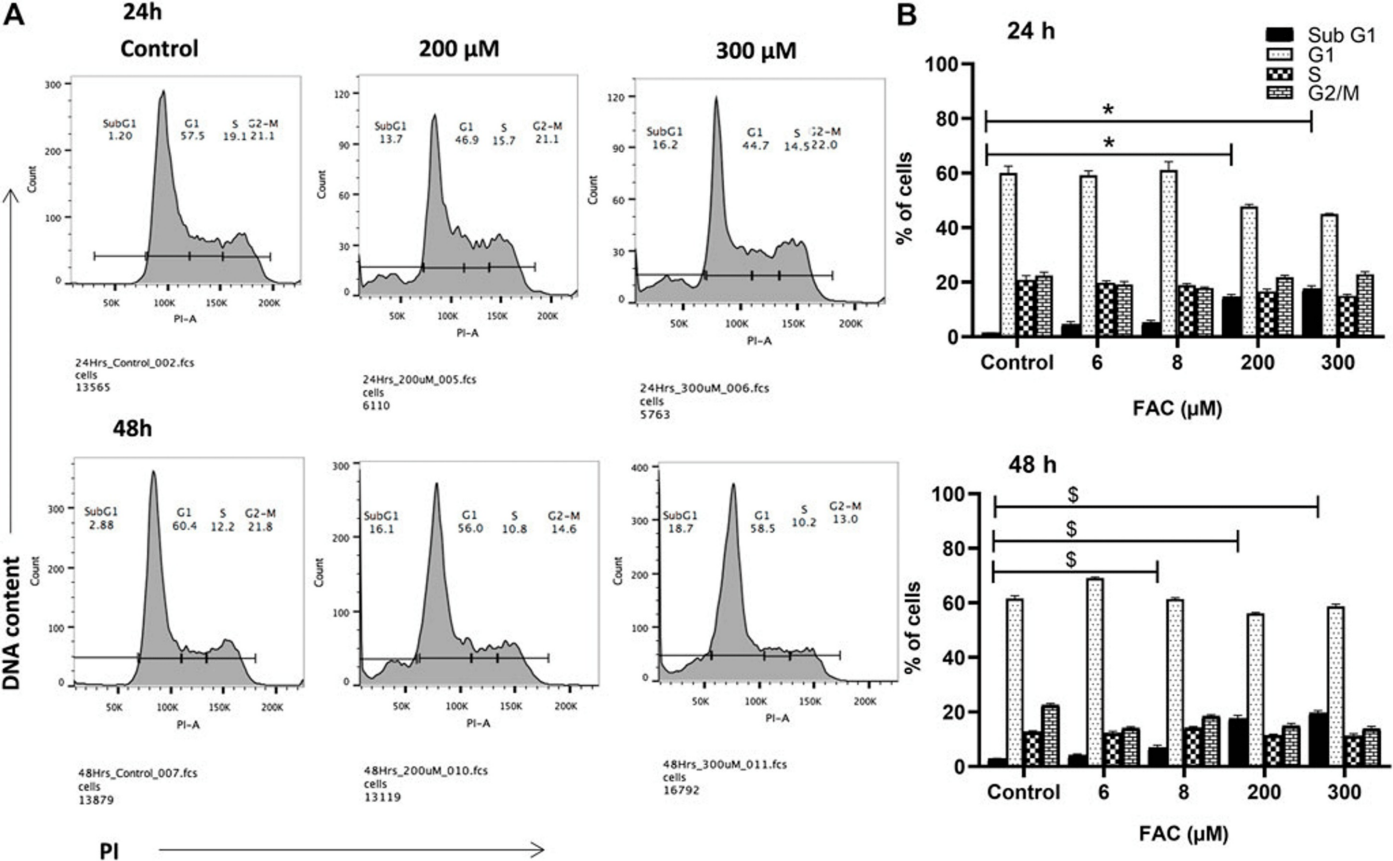
Description of cell cycle alteration in ferric ammonium citrate (FAC)-treated chondrocytes by Karim et al., 2022. Treatment of cells with FAC at 200 µM and 300 µM for 24–48 hours resulted in quantitative alterations of the cell cycle phase distribution, as assessed by flow cytometry, demonstrating iron-induced cytostatic effects. Image by Karim et al., 2022 [9]. Reproduced with open access permissions under the terms and conditions of the Creative Commons Attribution (CC BY) license (https://creativecommons.org/licenses/by/4.0/).

β-thalassemia is a genetic disorder of the blood where the production of the beta chains of hemoglobin is reduced or abolished, leading to long-term anemia and repeated blood transfusions [5,6]. In one research article that particularly addressed the neurological complications of beta-thalassemia, iron overload was quoted as a possible cause of numerous such complications, including nervous system complications [7]. The study also highlighted the propensity for malignancies among thalassemia patients, highlighting the need for caution in monitoring and controlling the iron status of such patients [8]. In addition to organ damage, iron overload was determined to disrupt cellular processes such as the cell cycle, where it displayed frank cytotoxic effects of iron accumulation on proliferative capacity (Figure 2) [9].

Clonal hematopoiesis of indeterminate potential (CHIP) is a condition that occurs with age as a result of somatic mutations within hematopoietic stem cells, leading to the emergence of somatically mutated blood cell clones [10]. CHIP has also been associated with a high risk of hematologic malignancy as well as cardiovascular disease [10]. The interplay between CHIP and iron overload is an emerging area of interest since both diseases intersect in common pathways that involve oxidative stress and inflammation and may increase the risk of cancer development in aging cohorts [11].

This review aims to explore the interface between iron overload, aging, and cancer, with particular interest in lessons from transfusion-dependent conditions such as beta-thalassemia. By an exploration of the pathways through which iron overload causes carcinogenesis and its role in aging-related factors such as CHIP, we aim to provide a profound understanding of risk and to inform monitoring and intervention strategies within populations at risk.

## Review

Iron metabolism and dysregulation in aging

Iron is tightly regulated under normal physiological conditions by a dynamic equilibrium between intestinal absorption, ferritin storage, and macrophage recycling [12]. Aging, nonetheless, is associated with stepwise accumulation of total body iron due to augmented cumulative absorption, reduced menstruation in females, and defective utilization [13]. 

Research has demonstrated that serum levels of ferritin and transferrin saturation are elevated with advancing age, particularly in men aged >65 years and in postmenopausal women [14]. Despite ferritin being a marker of iron storage, it is also a marker of inflammation and may hide mild iron loading [15].

**Figure 3:**
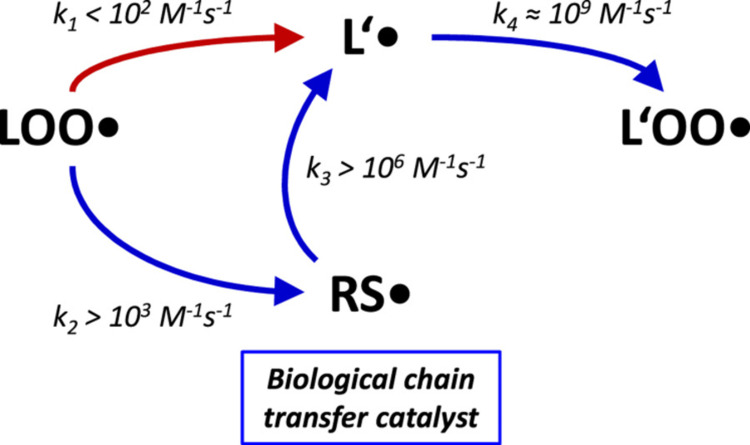
General rate constants and mitochondrial reaction rates of lipid peroxidation in the presence or absence of thiols. (A) The propagation step of the free radical lipid peroxidation chain reaction involves the attack of a lipid peroxyl radical (LOO•) towards a native lipid L′, resulting in a carbon-centered lipid radical (L’•). This reaction (red arrow) is the rate-limiting step of biological lipid peroxidation, as the subsequent reaction of the carbon-centered lipid radical (L’•) with molecular oxygen to yield a new lipid peroxyl radical (L’OO•) is much faster, by approximately a factor of 107 (ref. 18). As shown in this report, intramembrane thiol groups accelerate lipid peroxidation by offering a faster bypass to the commonly rate-limiting step via the formation of thiyl radicals (RS•). The slowest of the involved bypass rate constants is at least one order of magnitude higher than the commonly rate-limiting attack on L’ (k2 > 103 M−1s−1 versus k1 < 102 M−1s−1). Beyond their catalytic acceleration of lipid peroxidation, thiyl radicals (RS•) are sufficiently reactive to induce the oxidation of membrane proteins (Protein•) and the isomerization of monounsaturated fatty acids towards trans-fatty acids via an addition-fragmentation mechanism (RS-MUFA•). Rate constants were adopted from the following sources: k1 < 102 M−1s−1 (ref. 18); k2 > 103 M−1s−1 (ref. 25); k3 > 106 M−1s−1 (ref. 24); k4 ≈ 109 M−1s−1 (ref. 18); k5 ≈ 103-105 M−1s−1 (ref. 26); k6 ≈ 2 × 105 M−1s−1 (ref. 21). (B) Relative reaction rates of mitochondrial lipid peroxidation determined from specific, applicable rate constants and substrate concentrations found in the inner mitochondrial membrane (IMM) of adult rat liver. Details are provided in Supplementary Discussion S2. (For interpretation of the references to color in this figure legend, the reader is referred to the Web version of this article.) Image Credit: Kunath et al., 2020 [18]. Reproduced with open access permissions under the terms and conditions of the CC BY-NC-ND 4.0 license (https://creativecommons.org/licenses/by-nc-nd/4.0/).

Interestingly, there is too much iron to be a bystander; it catalyzes Fenton chemistry and forms hydroxyl radicals that are harmful to lipids, proteins, and DNA [16]. The reaction participates in the pathogenesis of age-related diseases, including neurodegeneration, cardiovascular disease, and now, cancer [17]. This process has been modeled both structurally and kinetically, showing how thiyl radicals short-circuit the classic bottleneck of lipid peroxidation, thereby propagating mitochondrial oxidative damage (Figure 3) [18-22].

The iron build-up in the long term not only illustrates metabolic drift but may also provide a premalignant condition, particularly in the liver and bone marrow [23]. It is necessary to realize this alteration in iron metabolism for the identification of susceptibility to cancer in the aged population, especially those with comorbidities exacerbating iron dysregulation.

Mechanisms of iron-induced carcinogenesis

**Figure 4:**
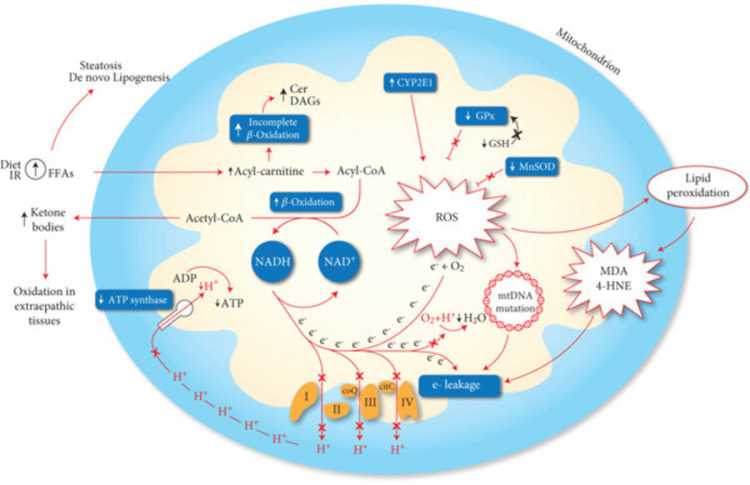
Mechanisms of mitochondrial dysfunction involved in the production of oxidative stress. An increase of mitochondrial beta-oxidation activity, due to a lipid overload, may induce an impairment of the electron transport chain, resulting in an “electron leakage.” The reaction between oxygen and protons catalyzed by cytochrome C oxidase (VI complex) is impaired, and electrons may interact directly with oxygen, forming ROS. Furthermore, the generation of mitochondrial membrane potential is reduced following the reduction of proton extrusion from the matrix, weakening the activity of ATP synthase. ROS production may exacerbate the mitochondrial dysfunction due to electron leakage following the generation of mtDNA mutation and highly reactive aldehydes (MDA, 4-HNE) produced through lipid peroxidation. Mitochondrial CYP2E1 is a direct source of ROS. A reduction of antioxidant mechanisms, as GPx and MnSOD was also observed in the NASH model. At last, the incomplete suboptimal oxidation of acyl-carnitine leads to the accumulation of lipotoxic intermediates (Cer, DAGs), which can act as an inflammatory intermediate altering the insulin signaling. 4-HNE: 4-hydroxy-2-nonenal; Cer: ceramides; CYP2E1: cytochrome P450 2E1; DAGs: diacylglycerols; FFAs: free fatty acids; GPx: glutathione peroxidase; GSH: glutathione; MDA: malondialdehyde; MnSOD: manganese superoxide dismutase; ROS: reactive oxygen species. Image Credit: Masarone et al., 2018 [26]. Reproduced with open access permissions under the terms and conditions of the CC BY 4.0 license (https://creativecommons.org/licenses/by/4.0/).

Iron plays a two-sided function in cell biology: it acts as an obligatory cofactor and possesses pro-oxidant activity if deregulated. Free iron catalyzes the Fenton reaction, generating highly reactive hydroxyl radicals in the context of iron overload that cause DNA, protein, and lipid damage, increasing mutagenesis and carcinogenesis [24]. The liver, in particular, is thereafter a target of choice due to its pivotal role in iron storage, and hepatocellular carcinoma caused by iron overload has been extensively reported in hemochromatosis and transfusional iron overload [2]. Animal models confirm that excess iron increases the incidence of spontaneous liver tumors, and hepatic iron concentration correlates positively with tumor mass [25]. A schematic representation of such mechanisms of mitochondrial dysfunction is presented in Figure 4 [26].

DNA damage due to oxidative stress is a primary oncogenic process initiated by excessive iron, and 8-hydroxy-2′-deoxyguanosine (8-OHdG) is a significant biomarker of such damage [27,28]. It has been proven that serum levels of 8-OHdG are significantly increased in patients with iron overload, up to 1.7-fold elevated in β-thalassemia patients compared to normal controls [29]. These iron-induced oxidative lesions can potentially lead to point mutations, deletions, and chromosomal instability, all of which converge to malignant transformation. Iron overload also suppresses DNA repair pathways and triggers epigenetic changes leading to oncogene activation and silencing of tumor suppressors [30].

Other than direct DNA damage, iron is also involved in establishing a pro-tumorigenic microenvironment through chronic inflammation and immune modulation. Iron triggers pro-inflammatory cytokine release, e.g., interleukin-6 (IL-6) and tumor necrosis factor-alpha (TNF-α), both implicated in cancer formation [31]. In older adults, this inflammatory response can become intertwined with immunosenescence, attenuating tumor immunity and enabling neoplastic escape. High iron has also been shown to induce macrophage polarization to a tumor-associated (M2-like) phenotype, further contributing to an immunosuppressive environment [32].

Iron also supports tumor growth through the facilitation of proliferation and angiogenesis. Transferrin receptors have a tendency to overexpress cancer cells to increase iron uptake, and this overexpression is associated with aggressive phenotypes of breast, liver, and pancreatic cancers [33,34]. The expression of transferrin receptors in tumor tissues was significantly elevated compared to that in normal tissues in hepatocellular carcinoma patients in one study [35,36]. Iron deficiency provokes angiogenesis by stabilizing hypoxia-inducible factor-1α (HIF-1α), which leads to overexpression of vascular endothelial growth factor (VEGF) and thus neovascularization [37].

Iron overload in transfusion-dependent disorders

Transfusion-dependent anemias such as β-thalassemia major, sickle cell disease (SCD), and myelodysplastic syndromes (MDS) require continuous red blood cell (RBC) transfusions to manage chronic anemia [38]. Each unit of transfused RBCs adds approximately 200-250 mg of iron to the body, and in the absence of any physiological mechanism of iron excretion, patients are at significant risk of iron overload [39].

In β-thalassemia major, patients begin transfusion treatment early in life and, consequently, accumulate iron eventually in significant quantities [40]. In accordance with one research, 87.4% of β-thalassemia major patients presented with serum ferritin levels greater than 1,000 ng/mL, with an average of 2,767.52 ng/mL, showing inadequate chelation and at risk of developing complications due to iron overload [40,41].

Iron overload in patients who are transfusion-dependent is not limited to β-thalassemia. In older patients, 30% of MDS patients become transfusion-dependent, leading to iron accumulation that can hasten the progression of disease as well as leukemic transformation [41].

**Figure 5:**
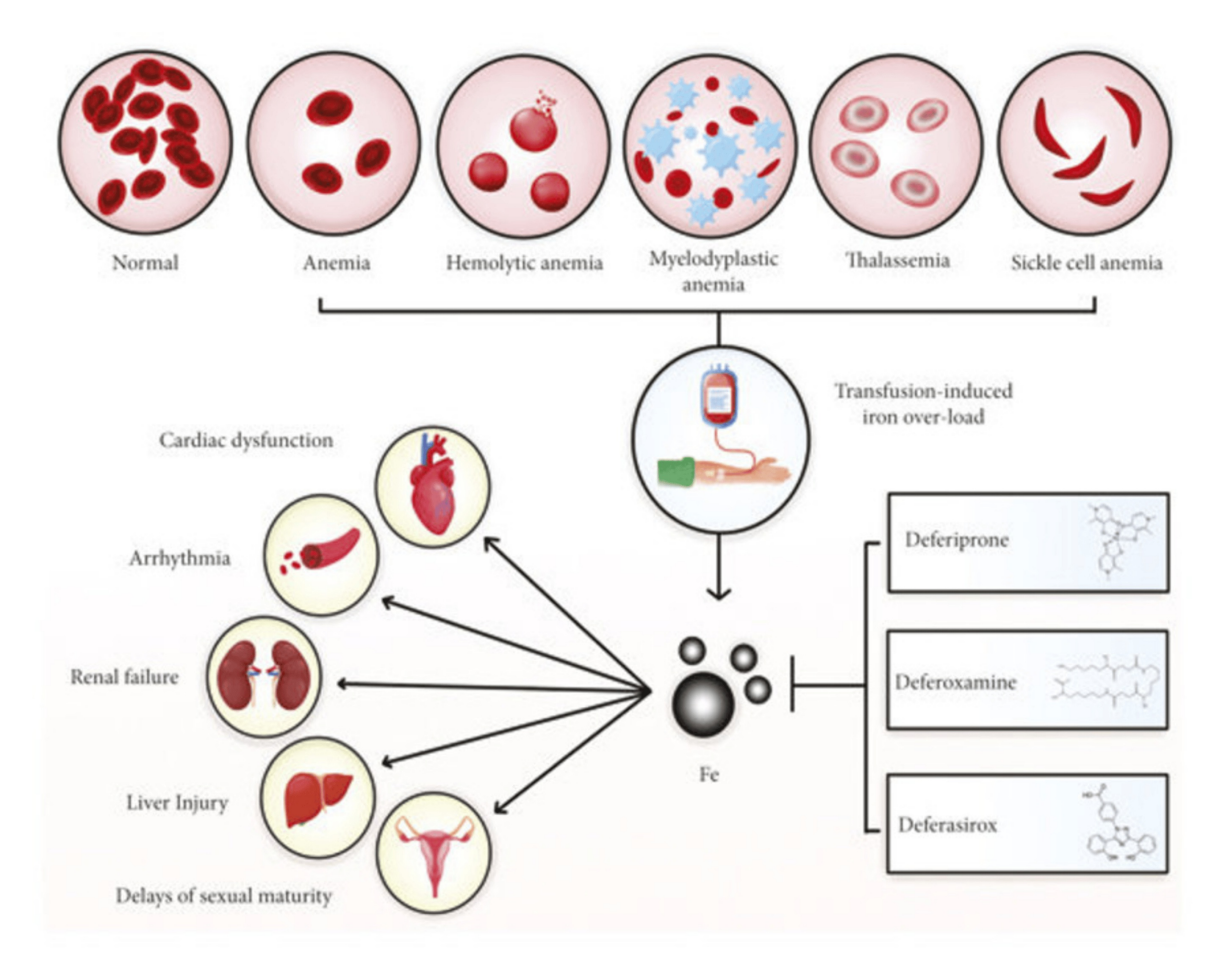
Deferoxamine, deferiprone, and deferasirox effects on transfusion-induced iron overload. Patients with aplastic anemia, hemolytic anemia, myelodysplastic anemia, thalassemia, and sickle cell anemia become transfusion dependent. Iron toxicity leads to free radical production, which causes severe side effects, including cardiac dysfunction, arrhythmia, renal failure, kidney damage, and delays in sexual maturity. Iron chelators can enter cells, bind free iron, and remove it from the body, thus inhibiting iron toxicity. Image Credit: Entezari et al., 2022 [43]. Reproduced with open access permissions under the terms and conditions of the CC BY 4.0 license (https://creativecommons.org/licenses/by/4.0/).

Iron overload pathophysiology is defined by the saturation of the principal iron transport protein, transferrin, with the consequential appearance of non-transferrin-bound iron (NTBI) in plasma. NTBI is readily taken up by parenchymal cells, leading to the generation of reactive oxygen species (ROS) and consequently cellular damage, particularly in the liver, heart, and endocrine glands [42]. A schematic representation of these mechanisms and the therapeutic application of iron chelators is provided in Figure 5 [43].

Therapy for transfusion-dependent iron overload, on the other hand, is primarily iron chelation therapy. The deferoxamine, deferiprone, and deferasirox agents have been shown to decrease serum ferritin and hepatic iron stores significantly, thus preventing organ damage [44].

Continuous surveillance of the iron content is important in these patients. Magnetic resonance imaging (MRI) methods, including T2* imaging, offer non-invasive tools to measure cardiac and hepatic iron deposition that help in the initiation and modulation of chelation therapy [45].

Clonal hematopoiesis and cancer risk

Clonal hematopoiesis (CH) is an age-related process that is manifest by the amplification of somatically mutated hematopoietic stem cells. CH occurs more frequently with increasing age, being present in less than 1% of people under the age of 40 years but rising to 10-20% in people older than 70 years [46]. A total of 455 participants in a study comparing whole-exome sequencing data in 12,380 people had CH, which indicates a high correlation with aging [47].

CH is associated with an increased risk for hematologic malignancies. The patients with CH have a 12.9 hazard ratio for hematologic cancer development compared to those without CH [47]. Furthermore, the presence of more than one CH mutation creates an even higher risk, and the hazard ratio is 42.2 for patients having more than 250 somatic single-nucleotide variants [47].

Exposure to cytotoxic treatments impacts CH dynamics. In treated individuals with high-grade ovarian cancer, CH with mutation-induced CH was observed in 35% of the patients, correlating with longer exposures to prior PARP inhibitor treatment [48]. The clones of therapy-related CH have an increased risk of therapy-related myeloid neoplasms [48].

**Figure 6:**
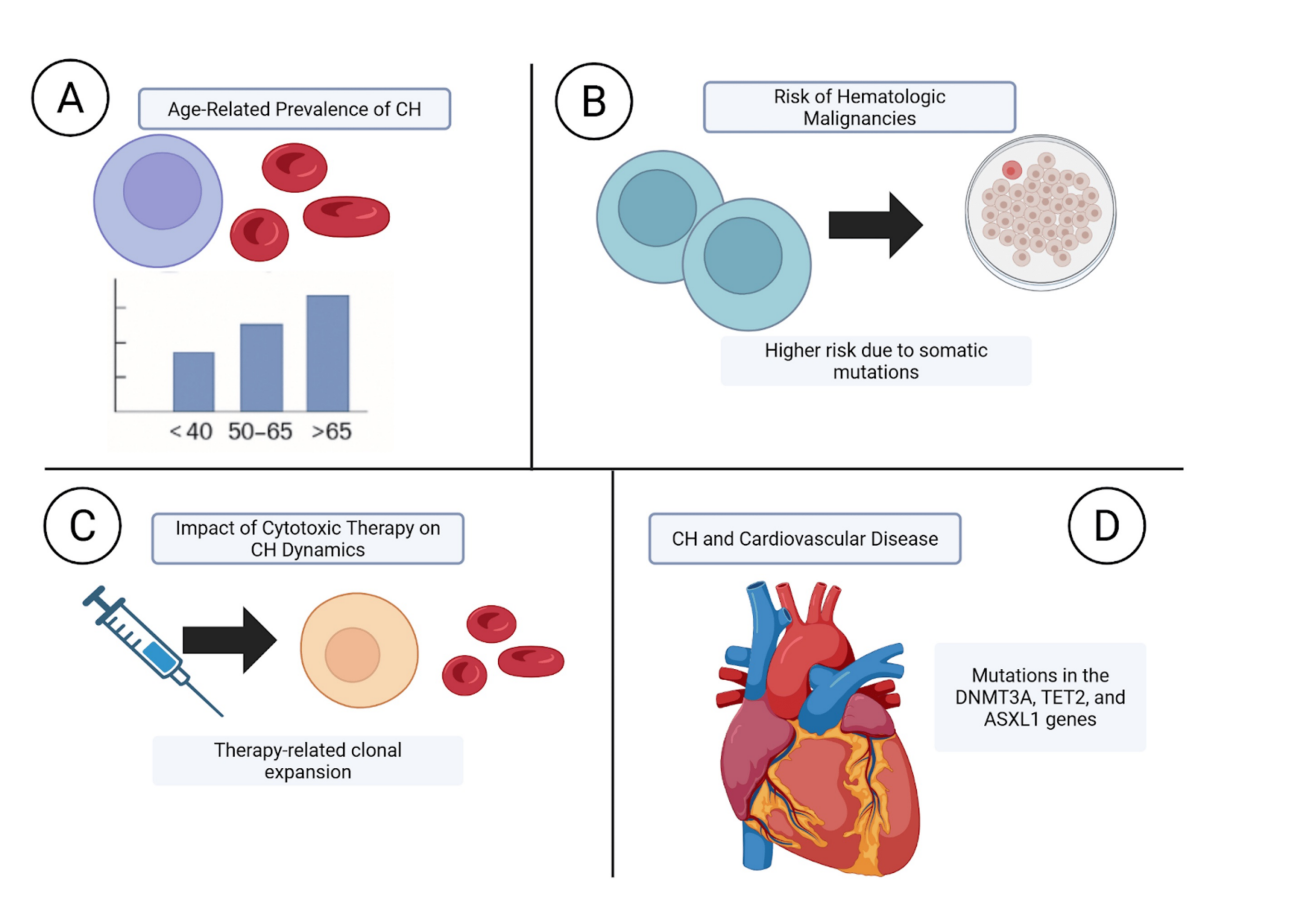
(A) This panel illustrates the increasing prevalence of CH with advancing age. (B) Depicts how mutated hematopoietic stem cells can expand clonally, leading to an elevated risk of hematologic cancers. (C) Highlights the influence of cytotoxic treatments, such as PARP inhibitors, on CH dynamics. (D) Demonstrates the link between CH-associated mutations (e.g., DNMT3A, TET2, ASXL1) and cardiovascular risk. Image created by Quang D. La using BioRender.

CH also affects outside hematologic malignancies. A study has demonstrated that patients with mutations in the DNMT3A, TET2, and ASXL1 genes have a 1.7- to 2.0-fold increased risk of acquiring coronary heart disease than those not harboring such mutations [49]. Further, the prevalence of CH in solid tumor-carrying patients is approximately 30%, suggesting a broader influence on cancer risk [49]. Figure 6 provides a comprehensive overview of the mechanisms and clinical implications of clonal hematopoiesis, including its age-related emergence, progression to hematologic malignancies, impact of cytotoxic therapies, and association with cardiovascular disease.

Iron chelation therapy-efficacy, adherence, and clinical outcomes

Iron chelation therapy (ICT) is required to treat transfusional iron overload in β-thalassemia major and myelodysplastic syndromes (MDS) [39]. Deferoxamine (DFO), administered by subcutaneous infusion for 8-12 hours daily, has been found to reduce hepatic iron concentration and serum ferritin levels [39]. Its rigid administration schedule, however, has caused poor compliance in patients [39].

Oral chelators like deferasirox (DFX) and deferiprone (DFP) have improved adherence as they are easier to administer [50]. In a study, adherence was 88.3% in those taking less than five tablets a day, increasing to 94.4% in those taking over 10 tablets per day [51]. ICT adherence correlates with significantly reduced levels of serum ferritin and with reduced risk of complications, including liver disease and cardiac disease [52].

Despite the benefits, ICT side effects can impact compliance. An association with gastrointestinal symptoms of nausea and vomiting as well as potential renal damage as increased serum creatinine level in about one-third of the patients has been seen [53]. The only serious side effect of deferiprone is agranulocytosis, a fatal disease that requires white blood cell counts to be monitored on a regular basis [54].

**Figure 7:**
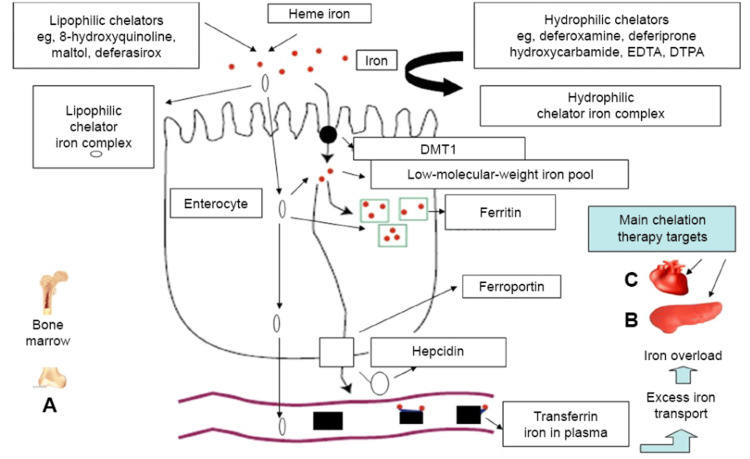
Iron-absorption and iron-overload mechanisms in non-transfusion-dependent thalassemias: the role of chelators and chelating drugs. Notes: Mechanism of iron absorption at the enterocyte using regulatory pathways of iron metabolism involving DMT1, hepcidin, ferroportin, and transferrin. Increased gastrointestinal iron absorption and iron overload are observed in non-transfusion-dependent thalassemias due to ineffective erythropoiesis in the bone marrow (A). The level of increased iron absorption depends on the form and quantity of iron present in the diet and other factors, such as the presence of natural or synthetic iron chelators in the gastrointestinal tract. Iron-chelating drugs and other chelators have variable pharmacological effects on iron absorption, with lipophilic chelators causing an increase in iron absorption and hydrophilic chelators a decrease in iron absorption. Excess iron absorption causes iron overload and damage in the liver, the heart, and other organs. The liver is the main organ of excess iron deposition, whereas the heart is the most susceptible organ of iron toxicity as a result of iron overload from increased iron absorption. Iron overload in both the liver (B) and the heart (C) in non-transfusion-dependent thalassemia are the main target sites of chelation therapy. DMT1, divalent metal transporter 1; EDTA, ethylenediaminetetraacetic acid; DTPA, diethylenetriaminepentaacetic acid. Image Credit: Kontoghiorghe & Kontoghiorghes, 2016 [56]. Reproduced with open access permissions under the terms and conditions of the Creative Commons Attribution - Non Commercial (unported, 3.0) License (https://creativecommons.org/licenses/by-nc/3.0/).

Combination regimens have been tried to increase efficacy. DFO in combination with DFP has shown Level A evidence to reduce myocardial iron loading, liver iron concentration, and serum ferritin [55]. Effects of newer regimens, especially combinations of drugs like DFX with DFO or DFP, remain a source of doubt [55]. A schematic overview of iron absorption, overload, and the mechanisms of action of iron chelators in thalassemia is shown in Figure 7 [56].

Careful follow-up and individually tailored treatment plans are needed to optimize ICT outcomes. MRI techniques, such as T2* imaging, allow non-invasive quantitation of cardiac and hepatic iron loading to guide chelation therapy initiation and titration [39]. Individually tailored treatment and monitoring for adverse reactions can improve compliance and reduce the risk of iron overload complications [39].

Iron overload and cancer risk

Iron overload has been associated with an increased risk of cancer, particularly hepatocellular carcinoma (HCC). Hereditary hemochromatosis patients have a 20-200 times higher risk of developing HCC than the general population [57]. This risk is primarily because of iron overload in the liver, leading to cirrhosis and subsequently malignant transformation [57]. In addition, iron-induced oxidative stress may cause DNA damage, making carcinogenesis more favorable [58].

Apart from liver cancer, iron overload has been implicated in increased risks for other cancers. A meta-analysis and systematic review revealed that increased body iron stores are linked to increased risks for colorectal, esophageal, and lung cancers. In particular, individuals with higher serum ferritin levels had a 1.33-fold increased risk of developing colorectal cancer [59]. The findings indicate the systemic influence of iron overload on cancer development.

Mechanistically, iron overload induces carcinogenesis through the formation of reactive oxygen species (ROS). The free iron-catalyzed Fenton reaction produces hydroxyl radicals that can oxidize DNA, proteins, and lipids [58]. Oxidative stress not only mutates but also affects cell signaling pathways regulating proliferation and apoptosis [58]. Immune surveillance may also be compromised by iron overload and induce tumor growth [60].

**Figure 8:**
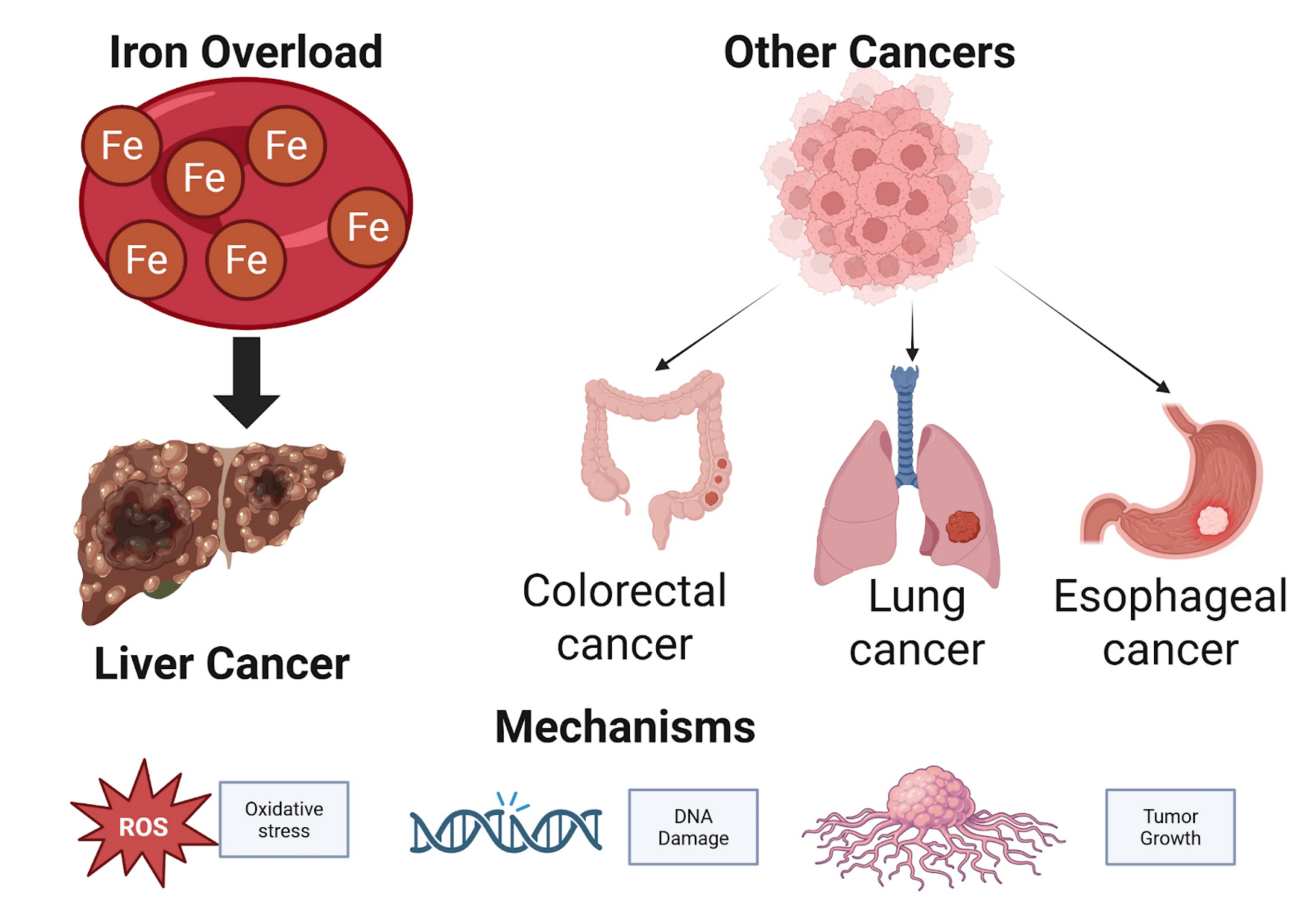
Visual representation of the link between iron overload and cancer. Excess iron, particularly in the liver, increases the risk of hepatocellular carcinoma (HCC) through mechanisms such as oxidative stress, DNA damage, and disrupted cell signaling. Elevated iron levels are also associated with heightened risks of colorectal, lung, and esophageal cancers, highlighting the systemic impact of iron-induced carcinogenesis. Image created by Quang D. La using BioRender.

In brief, iron overload is a proven risk factor for a wide array of cancers, and there is both epidemiologic and mechanistic evidence for its role in carcinogenesis (Figure 8). An understanding of the mechanisms whereby excess iron promotes tumor growth is critical to the planning of preventive and therapeutic interventions [58]. Additional studies are needed in order to determine the potential benefits of iron reduction therapies for cancer prevention and treatment [59].

Iron metabolism and aging

Aging is associated with alterations in iron homeostasis, leading to increased iron accumulation in various tissues. There has been a report that serum ferritin concentrations rise with age and reflect increased body stores of iron [61]. Accumulation is most extensive in the liver, heart, and brain organs susceptible to iron-induced oxidative damage [61]. Dysregulation of iron metabolism in aging leads to age-related disease pathogenesis, such as neurodegenerative disorders and cancer [61].

Mitochondrial dysfunction is a hallmark of aging, and iron is a determinant of mitochondrial health. Excess mitochondrial iron can catalyze the formation of reactive oxygen species (ROS), generating oxidative damage to mitochondrial DNA and proteins [62]. Such oxidative stress impairs mitochondrial function, inducing cellular senescence and aging phenotypes [63]. Moreover, iron-induced mitochondrial dysfunction has been implicated in the pathogenesis of age-related neurodegenerative diseases such as Parkinson's and Alzheimer's [64].

Systemic regulation of iron is primarily controlled by the hormone hepcidin, whose levels decrease with age. Older age is characterized by decreased levels of hepcidin, leading to increased intestinal iron absorption and release of iron from macrophages, exacerbating iron accumulation [65]. This dysregulation is responsible for the pro-inflammatory condition that is present in aging because increased iron activates the generation of pro-inflammatory cytokines [65]. In turn, chronic inflammation further disturbs iron homeostasis, creating a vicious cycle that increases aging processes [66].

Interventions that target iron metabolism have been demonstrated to be efficacious in the prevention of age-related pathologies. Caloric restriction, an established anti-aging intervention, has been reported to reduce iron overload and oxidative stress in animal models [67]. Pharmacologic agents such as iron chelators are being explored for their therapeutic potential in preventing iron-induced oxidative damage in aging tissues [56]. These interventions target restoring iron homeostasis, thereby improving mitochondrial function and avoiding the risk of age-related diseases [56].

## Conclusions

Iron overload is an underemphasized major cause of aging-related pathology and malignancy, especially in transfusion-dependent patient populations such as β-thalassemia and myelodysplastic syndromes. As depicted, the intersection of dysregulated iron metabolism, clonal hematopoiesis, and cellular senescence allows for the establishment of cancer. Enhanced disease surveillance with refinements in iron chelation therapy and imaging is feasible, but further integration of personalized screening and prevention into management is paramount. Understanding and preventing iron-induced biological damage in the aging body may yield powerful new approaches to reducing cancer risk and improving healthspan in vulnerable populations.

## Figures and Tables

**Figure 9:**
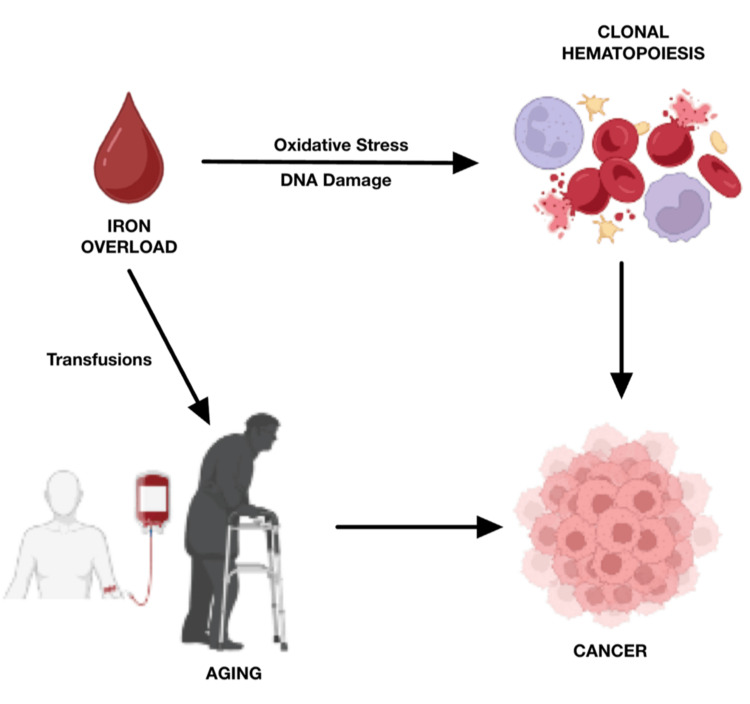
Iron Overload, Clonal Hematopoiesis, and Cancer Risk in Aging and Transfusion-Dependent-Population. Created by Marc Faltas using BioRender.

## Appendices

Figure 9 showcases the graphical abstract of this literature review.
